# A newer and broader definition of burnout: Validation of the "Burnout Clinical Subtype Questionnaire (BCSQ-36)"

**DOI:** 10.1186/1471-2458-10-302

**Published:** 2010-06-02

**Authors:** Jesús Montero-Marín, Javier García-Campayo

**Affiliations:** 1 Department of Psychiatry. University of Zaragoza. Zaragoza. Spain; 2 Psychiatry Service. Miguel Servet Hospital. Zaragoza. Spain

## Abstract

**Background:**

Burnout syndrome has been clinically characterised by a series of three subtypes: frenetic, underchallenged and worn-out, with reference to coping strategies for stress and frustration at work with different degrees of dedication. The aims of the study are to present an operating definition of these subtypes in order to assess their reliability and convergent validity with respect to a standard burnout criterion and to examine differences with regard to sex and the temporary nature of work contracts.

**Method:**

An exploratory factor analysis was performed by the main component method on a range of items devised by experts. The sample was composed of 409 employees of the University of Zaragoza, Spain. The reliability of the scales was assessed with Cronbach's α, convergent validity in relation to the Maslach Burnout Inventory with Pearson's *r*, and differences with Student's t-test and the Mann-Whitney U test.

**Results:**

The factorial validity and reliability of the scales were good. The subtypes presented relations of differing degrees with the criterion dimensions, which were greater when dedication to work was lower. The frenetic profile presented fewer relations with the criterion dimensions while the worn-out profile presented relations of the greatest magnitude. Sex was not influential in establishing differences. However, the temporary nature of work contracts was found to have an effect: temporary employees exhibited higher scores in the frenetic profile (*p *< 0.001), while permanent employees did so in the underchallenged (*p *= 0.018) and worn-out (*p *< 0.001) profiles.

**Conclusions:**

The classical Maslach description of burnout does not include the frenetic profile; therefore, these patients are not recognised. The developed questionnaire may be a useful tool for the design and appraisal of specific preventive and treatment approaches based on the type of burnout experienced.

## Background

Burnout syndrome has been described as a prolonged response to chronic emotional and interpersonal stressors on the job, determined by the dimensions of exhaustion, cynicism, and inefficacy [1]. Exhaustion is described as the feeling of not being able to offer any more of oneself at an emotional level; cynicism as a distant attitude towards work, the people being served by it and colleagues; and inefficacy as the feeling of not performing tasks adequately and of being incompetent at work. In general terms, burnout is the body's response to the failure of the coping strategies that individuals typically utilise to manage stressors at work [2].

Despite the various definitions of the syndrome presented in the literature, burnout has traditionally been described as a relatively uniform entity in all individuals, with more or less consistent aetiology and symptoms [3]. Nevertheless, clinical and therapeutic experience refutes this hypothesis, resulting in the need to characterise the different types of burnout in order to adjust lines of therapeutic action for more effectiveness. Farber [4] has proposed a preliminary typology with three syndrome profiles (frenetic, underchallenged, and worn-out); this typology may allow for the development of more specific treatments [3]. Based on Farber's clinical and phenomenological work [3-9], our group [10] has theoretically systematised this typology, specifying the properties on which the profiles are based and establishing a classification criterion that coherently expresses the proposal in its entirety.

The frenetic type [10] comprises a category of highly applied and committed individuals who are characterised by the investment of a substantial amount of time and effort in their dedication to work. The characteristics of individuals with this clinical profile are a high degree of involvement, in the form of increasingly greater efforts in the face of difficulties; grandiosity, in the sense of great ambition and need for achievements; and overload, as feeling of being overwhelmed caused by the neglect of their own needs (health and personal life) in an attempt to satisfy work requirements. The underchallenged type [10] is described as comprising individuals who have no interest in their work and perform tasks in a superficial manner because they lack challenges, motivation or desire for engagement. The characteristics of this profile are indifference, as a means of working superficially and without interest; lack of development, owing to the dissatisfaction of one's talents remaining unacknowledged until other employment options are contemplated; and boredom, in the sense of experiencing work as a monotonous and routine event. The worn-out type [10] comprises individuals whose level of involvement in their work is reduced to the point where they disregard the responsibilities of their position. The characteristics of this profile are neglect, as a lack of involvement in the work tasks to the point of giving up in the face of difficulty; lack of acknowledgement, as the feeling of not having their efforts and dedication recognised; and lack of control, as the desperation caused by their lack of control over the results of their actions at work.

The classification criterion (dimension on which differentiation is based) is the degree of dedication [10], specifically reflected in the values of involvement, indifference and neglect, which are the methods of coping with stress and frustration at work. However, affected individuals may defy this classification [4] by fluctuating between the three profiles [8] or by gradually evolving from one profile into another over time as their dedication diminishes [5,10].

In a previous exploratory study carried out by our group [11], associations between burnout subtype characteristics and variables such as dissatisfaction with job and organisation, severity of burnout (measured with an instrument based on the definition of Maslach and Jackson [12]), and the physical, psychological and social consequences of burnout (according to Moreno et al.'s model [13]) were found. Moreno et al.'s model, based on the definition proposed by Schwab et al. [14], has been replicated by our group with consistent results [15].

Within this framework, the main aim of the current study was to construct a questionnaire that would allow the clinical profiles reflected in the previously described conceptual structure to be operationalised. We also evaluated the internal consistency of the constituent scales and subscales as well as their convergent validity with regard to a standard burnout criterion. Lastly, we examined the potential differences caused by sex and the temporary nature of work contracts.

## Methods

We used the correlational method with a cross-sectional design. The measurements were obtained by means of the self-assessment technique using a questionnaire. All participants provided their informed consent.

### Participants

The study population consisted of the employees of the University of Zaragoza who were employed in January 2008 (N = 5,493). The sample size was calculated for a 95% confidence interval with a 3.5% error, assuming the prevalence of burnout to be 18%, according to previous studies on the general population [2,16]. The calculation yielded a result of 427 subjects. The response rate expected in web-mail surveys is about 27% [17,18]. Therefore, 1,600 subjects were chosen by means of random stratified sampling with proportional allocation depending on occupation from an alphabetical list of the entire workforce. The final sample was composed of 409 participants, with a response rate of 25.6%. The response rate was distributed as follows: 19.3% teaching and research staff, 36.5% administration and service personnel, 25.8% fellows. The number of participants exceeded the contruct validity evaluation criterion [19], resulting in a sample that was psychometrically adequate for the study.

The mean age of participants was 40.51 years (SD = 9.09). Of the participants, 44.4% were males. In terms of job position, 42.9% of the subjects were teaching and research staff members, 46.9% were administration and service personnel and 10.2% were fellows. Of the sample, 21.9% were not in a stable relationship, and 49.9% had children. In terms of length of employment, 18.5% had been working at the university for less than 4 years, with 44.6% working between 4 and 16 years and 36.9% for more than 16 years. The income distribution was as follows: 31.1% had a monthly income of less than €1,200, with 42.1% earning €1,200-2,000 per month and 26.8% earning more than €2,000. Nearly 67% of the participants did not take sick leave in the previous year. Of the subjects, 63.6% were permanent employees and 93.8% worked full time.

### Tools

Subjects were first asked questions concerning general socio-demographic and work-related aspects for the purpose of providing a description of the participating sample and carrying out the previously mentioned contrasts. They were then presented with a self-administered questionnaire that consisted of 72 items, 8 for each of the 9 characteristics included in the previously described model. The items were developed by a group of experts who attempted to include the main characteristics of the reference domain by means of consensus [10]. The wording of the items was guided by a table of content specifications, which enabled the fit, conceptual validity and representative nature of the proposal to be assured. This initial battery of instruments was overdimensioned in order to select the items with the best psychometric properties based on the Classical Theory of Tests [20-22]. Subjects indicated their degree of agreement with each of the statements presented using a Likert-type scale with 7 response options, scored from 1 (totally agree) to 7 (totally disagree).

To conclude, subjects were presented with the Maslach Burnout Inventory-General Survey (MBI-GS) [23] in the validated Spanish language version adapted by Bresó, Salanova and Schaufeli [24]. This adaptation consists of 15 items grouped into three dimensions. Responses were arranged in a Likert-type scale with 7 options, scored from 0 (never) to 6 (always). The exhaustion dimension (comprising 5 items such as "I feel emotionally drained from my work") achieved α = 0.92 in our study. The cynicism dimension (comprising 4 items such as "I've become more callous toward people since I took this job") obtained α = 0.92. The efficacy dimension (consisting of 5 items such as "I deal very effectively with the problems of my work") achieved α = 0.82.

### Data analysis

From the proposed items, we selected those with the best discrimination coefficient in their respective domain [20-22]. The factor structure of the scales was tested by means of Exploratory Factor Analysis (EFA), following the main component method with varimax orthogonal rotation. In order to confirm the legitimacy of the analysis, we confirmed that the KMO index had a value >0.70 and that Bartlett's sphericity test provided a significant result. The number of components was decided using Kaiser's criterion [25], which requires eigenvalues greater than one, in addition to Cattel's scree test [26] on the sedimentation graph. In addition, the criterion of factorial weight >0.5 was used to determine which items were allocated to a specific factor [19]. The percentage variance explained in each item by its pertinence factor was calculated with *c*^*2 *^communality values, the reliability of scales and subscales with Cronbach's α and relation to the criterion with Pearson's r. Contrast tests were calculated with Student's t-test for independent measurements or through *z *values associated with the Mann-Whitney U test (depending on the normality hypothesis). Data analysis was performed with the SPSS version 15 statistics software package.

### Procedure

An e-mail explaining the aims of the research was sent to the selected subjects. The e-mail contained a link to an online questionnaire and two access passwords for subjects to complete the questionnaire during the month of February 2008. As a token of appreciation for their collaboration in the study, participants received a report with their score from the questionnaire and its interpretation. This project was approved by the Ethics Committee of Aragon.

## Results

The following paragraphs present the results obtained from the selected items according to the method previously described based on the Classical Theory of Tests.

### Exploratory Factor Analysis (EFA)

The distribution of items on the frenetic scale allowed the use of the EFA (KMO = 0.83; Bartlett *p *< 0.001). This analysis provided an unforced solution for three factors. The first of these (ambition) presented an eigenvalue of 4.37 (36.44% variance); the second (overload) had an eigenvalue of 2.41 (20.09%); and the third (involvement) exhibited an eigenvalue of 1.67 (13.94%). The three factors exceeded Kaiser's criterion and the scree test allowed the solution to be accepted as adequate. In total, 70.47% of the variance was explained.

The distribution of items on the unchallenged scale permitted EFA (KMO = 0.92; Bartlett *p *< 0.001), which provided an unforced solution for three factors. The first of these (indifference) presented an eigenvalue of 6.91 (57.57%); the second (lack of development) had an eigenvalue of 1.40 (11.66%); and the third (boredom) exhibited an eigenvalue of 1.01 (8.34%). The three factors exceeded Kaiser's criterion, and the sedimentation graph slope became gentle for these three factors. The solution explained 77.57% of the total variance.

The distribution of items on the worn-out scale made EFA possible (KMO = 0.86; Bartlett *p *< 0.001). EFA provided an unforced solution for three factors. The first of these (lack of acknowledgement) presented an eigenvalue of 4.89 (40.76% variance); the second (neglect) had an eigenvalue of 2.44 (20.34%); and the third (lack of control) exhibited an eigenvalue of 1.23 (10.21%). The three factors exceeded Kaiser's criterion, and the scree test offered a structure for the three factors. This model explained 71.31% of the total variance.

Table 1 shows the rotated factor solution and descriptive statistics of the items belonging to the three scales (see Additional file 1 for item content). The responses to the items of the involvement and neglect factors were more extreme and, in particular, less variable than the others. The discrimination coefficients show raised positive values in the belonging factor, while they were lower, albeit adequate, in the total scale of the corresponding profile. All communality values were adequate.

**Table 1:** Factor weighting and descriptive statistics of the BCSQ-36

Scales/Items	Factor Weighting						
					
*Frenetic*	Ambition	Overload	Involvement	M	SD	discr F/S	***c***^**2**^
No. 1	**0.89**	0.13	0.12	3.72	1.42	0.81/0.60	0.82
No. 4	**0.81**	0.17	0.16	3.91	1.38	0.73/0.60	0.72
No. 7	**0.83**	0.16	0.16	4.01	1.35	0.75/0.61	0.74
No. 10	**0.84**	0.10	0.16	4.00	1.38	0.75/0.58	0.75

No. 2	0.10	**0.87**	0.05	3.79	1.53	0.77/0.52	0.77
No. 5	0.18	**0.83**	0.04	3.18	1.59	0.73/0.55	0.73
No. 8	0.11	**0.85**	0.04	3.37	1.55	0.73/0.51	0.74
No. 11	0.13	**0.77**	0.03	3.79	1.44	0.63/0.46	0.61

No. 3	0.15	0.10	**0.84**	5.08	0.99	0.70/0.45	0.74
No. 6	0.10	0.17	**0.70**	4.96	1.13	0.53/0.39	0.53
No. 9	0.17	0.01	**0.81**	4.95	0.95	0.66/0.39	0.69
No. 12	0.13	-0.12	**0.77**	4.68	1.14	0.59/0.30	0.63

*Underchallenged*	Indifference	L. Development	Boredom				

No. 13	**0.80**	0.27	0.24	2.65	1.46	0.79/0.70	0.77
No. 16	**0.79**	0.22	0.30	2.59	1.39	0.79/0.70	0.76
No. 19	**0.73**	0.38	0.30	2.90	1.55	0.76/0.77	0.77
No. 22	**0.84**	0.07	0.14	2.17	1.14	0.67/0.54	0.73

No. 14	0.14	**0.86**	0.22	3.72	1.66	0.79/0.66	0.82
No. 17	0.26	**0.74**	0.24	3.32	1.46	0.70/0.67	0.67
No. 20	0.21	**0.86**	0.20	4.03	1.61	0.80/0.68	0.82
No. 23	0.22	**0.71**	0.33	3.86	1.68	0.69/0.68	0.66

No. 15	0.24	0.24	**0.87**	3.01	1.53	0.85/0.73	0.87
No. 18	0.20	0.30	**0.84**	3.15	1.61	0.79/0.71	0.83
No. 21	0.38	0.30	**0.75**	3.03	1.56	0.82/0.78	0.80
No. 24	0.46	0.32	**0.62**	2.95	1.54	0.78/0.82	0.80

*Worn-out*	L. Acknowledgement	Neglect	L. Control				

No. 25	**0.81**	0.07	0.15	3.93	1.68	0.67/0.57	0.68
No. 28	**0.74**	0.19	0.25	4.68	1.69	0.67/0.65	0.64
No. 31	**0.88**	0.09	0.20	4.58	1.65	0.83/0.68	0.82
No. 34	**0.85**	0.08	0.28	4.50	1.60	0.81/0.71	0.81

No. 26	0.13	**0.79**	0.02	2.58	1.16	0.66/0.38	0.65
No. 29	0.09	**0.83**	0.22	2.53	1.07	0.74/0.49	0.75
No. 32	0.07	**0.87**	0.04	2.32	0.97	0.76/0.38	0.76
No. 35	0.07	**0.84**	0.08	2.65	1.06	0.72/0.39	0.71

No. 27	0.36	0.08	**0.75**	4.53	1.53	0.70/0.63	0.70
No. 30	0.43	-0.05	**0.71**	4.98	1.37	0.65/0.59	0.69
No. 33	0.18	0.32	**0.68**	3.83	1.50	0.55/0.57	0.60
No. 36	0.10	0.07	**0.84**	4.44	1.43	0.64/0.49	0.73

Extraction: main components. Rotation: varimax. discr F/S = discrimination factor/scale coefficient. *c*^*2 *^= comunalities.

### Scale and subscale descriptive statistics and reliability

Table 2 shows the descriptive statistics and reliability of the scales and subscales (calculated as the sum of the component items divided among their number). The highest mean (scalar) scores were those of the frenetic profile (Md = 4.12; SD = 0.80), followed by those of worn-out (Md = 3.79; SD = 0.90) and those of the underchallenged profile (Md = 3.12; SD = 1.15). Underchallenged was the profile that showed the greatest variability.

**Table 2:** Descriptive statistics and correlations between BCSQ-36 and MBI-GS dimensions

Scales/Subscales	M (SD)	*Frenetic*	Ambition	Overload	Involvement
*Frenetic*	4.12 (0.80)	(0.84)			
Ambition	3.91 (1.20)	0.79**	(0.89)		
Overload	3.53 (1.29)	0.74**	0.31**	(0.86)	
Involvement	4.92 (0.84)	0.59**	0.34**	0.12*	(0.80)

Exhaustion	2.39 (1.42)	0.30**	0.08	0.58**	-0.14**
Cynicism	2.07 (1.59)	-0.05	-0.08	0.21**	-0.35**
Efficacy	4.45 (1.01)	0.24**	0.26	0.09	0.45**

		*Underchallenged*	Indifference	L. Development	Boredom

*Underchallenged*	3.12 (1.15)	(0.92)			
Indifference	2.58 (1.20)	0.85**	(0.88)		
L. Development	3.73 (1.37)	0.85**	0.56**	(0.88)	
Boredom	3.04 (1.40)	0.90**	0.69**	0.64**	(0.92)

Exhaustion	2.39 (1.42)	0.39**	0.40**	0.38**	0.25**
Cynicism	2.07 (1.59)	0.66**	0.65**	0.60**	0.49**
Efficacy	4.45 (1.01)	-0.38**	-0.49**	-0.22**	-0.31**

		*Worn-out*	L. Acknowledgement	Neglect	L. Control

*Worn-out*	3.79 (0.90)	(0.87)			
L. Acknowledgement	4.42 (1.42)	0.86**	(0.88)		
Neglect	2.52 (0.90)	0.58**	0.25**	(0.86)	
L. Control	4.44 (1.17)	0.82**	0.57**	0.27**	(0.81)

Exhaustion	2.39 (1.42)	0.62**	0.49**	0.32**	0.59**
Cynicism	2.07 (1.59)	0.68**	0.59**	0.43**	0.53**
Efficacy	4.45 (1.01)	-0.43**	-0.23**	-0.55**	-0.29**

Values in parentheses of the diagnonal in each matriz are α coefficients. **p < 0.001. *p < 0.05.

As expected based on the nature of the factor analysis, the α coefficients obtained were good (all of which were >0.8). Each of the items contributed to raising the reliability of their factor as well as the total scale of their profile, except items 12 and 22, which raised the reliability of their factor but not that of their profile. Nevertheless, elimination of these items resulted in the same value for the general corresponding scales; therefore, they were not rejected.

### Convergent validity

Convergence values with the MBI-GS differed for each of the identified burnout types. The frenetic profile presented fewer relations with the criterion dimensions. The relations were moderate for exhaustion (*r *= 0.30; *p *< 0.001), insignificant for cynicism (*r *= -0.05; *p *= 0.352) and moderately low in a positive sense for efficacy (*r *= 0.24; *p *< 0.001). The underchallenged profile presented relations of the greatest magnitude. The relations were moderate for exhaustion (*r *= 0.39; *p *< 0.001), very high for cynicism (r = 0.66; p < 0.001) and moderate for efficacy in an inverse sense (*r *= -0.38; *p *< 0.001). The worn-out profile obtained the greatest relations with the criterion. The relations were very high for exhaustion (*r *= 0.62; *p *< 0.001) and cynicism (r = 0.68; p < 0.001), and moderately high for efficacy in a negative sense (*r *= -0.43; *p *< 0.001). Table 2 shows the descriptive statistics and the correlations between BCSQ-36 and MBI-GS dimensions.

### Differences owing to sex and the temporary nature of work contracts

Table 3 shows the descriptive statistics and results of contrast tests for the three profile scales and subscales. No significant differences by sex were found for any of the scales or subscales, but the temporary nature of work contracts was found to be a determinant. Temporary employees exhibited higher scores in the frenetic profile (*p *< 0.001), while permanent employees did so in the underchallenged (*p *= 0.018) and worn-out (*p *< 0.001) profiles.

**Table 3:** Descriptive statistics and BCSQ-36 scales depending on sex and temporary nature of work contracts

	M (n = 182)	F (n = 227)		P (n = 260)	T (n = 149)	
				
SCALES	M (SD)	M (SD)	t (p)	M (SD)	M (SD)	t (p)
Frenetic	4.15 (0.83)	4.09 (0.77)	0.76 (p = 0.445)	3.99 (0.74)	4.34 (0.84)	-4.21 (p < 0.001)
Underchallenged	3.25 (1.25)	3.02 (1.06)	1.95 (p = 0.053)	3.22 (1.12)	2.94 (1.18)	2.38 (p = 0.018)
Worn-out	3.79 (0.87)	3.79 (0.92)	0.02 (p = 0.987)	3.92 (0.86)	3.56 (0.93)	3.97 (p < 0.001)

SUBSCALES	Median (Q_1_-Q_3_)	Median (Q_1_-Q_3_)	z (p)	Median (Q_1_-Q_3_)	Median (Q_1_-Q_3_)	z (p)

-Ambition	4.00 (3.00-5.00)	3.75 (3.00-4.50)	-1.60 (p = 0.109)	3.50 (3.00-4.50)	4.25 (3.25-5.00)	-4.20 (p < 0.001)
-Overload	3.25 (2.75-4.50)	3.25 (2.50-4.31)	-0.65 (p = 0.514)	3.25 (2.75-4.25)	3.50 (2.75-4.62)	-1.45 (p = 0.147)
-Involvement	4.87 (4,50-5,25)	5.00 (4,50-5,25)	-1.46 (p = 0.144)	4.75 (4.25-5.25)	5.00 (4.75-5.75)	-4.04 (p < 0.001)

-Indifference	2.50 (1.75-3.25)	2.50 (1.75-3.00)	-1.10 (p = 0.272)	2.50 (1.75-3.25)	2.00 (1.50-3.00)	-2.97 (p = 0.003)
-L.Development	3.75 (3.00-4.75)	3.50 (3.00-4.50)	-1.16 (p = 0.247)	3.75 (3.00-4.62)	3.25 (2.50-4.62)	-1.76 (p = 0.077)
-Boredom	3.00 (2.00-4.25)	3.00 (2.00-3.75)	-1.90 (p = 0.057)	3.00 (2.25-4.00)	2.75 (1.75-3.75)	-3.01 (p = 0.003)

-L.Acknowledgement	4.50 (3.50-5.50)	4.25 (3.25-5.50)	-0.52 (p = 0.603)	4.50 (3.50-5.50)	4.00 (3.00-5.50)	-2.77 (p = 0.006)
-Neglect	2.50 (1.81-3.00)	2.75 (2.00-3.00)	-0.05 (p = 0.960)	3.00 (2.25-3.00)	2.25 (1.50-3.00)	-5.12 (p < 0.001)
-L.Control	4.37 (3.50-5.00)	4.50 (3.50-5.25)	-0.85 (p = 0.392)	4.50 (3.75-5.25)	4.25 (3.50-5.12)	-1.81 (p = 0.071)

M = male. F = female. P = permanent. T = temporary.

## Discussion

This study is the first with the aim of producing an operational concept of professional burnout that enables classification into clinical subgroups. This concept was a need felt by clinicians because not all individuals with burnout present the same characteristics and prognosis. Analysis of the selected items and resulting scales for each profile has confirmed the factor validity and high reliability of the model. All of the operational definitions were faithful to the meanings contained in the Farber's theory.

The frenetic scale was composed of the involvement, ambition and overload dimensions. The high scores and low variability obtained in the items belonging to the involvement factor suggest that these responses may be influenced by social desirability, an aspect that should be considered when establishing anchoring points on a scalar level in later studies. The frenetic profile generally presented significant relations with exhaustion and with efficacy in a positive sense. These subjects are affected by burnout, given that this is what they express in therapy sessions when manifesting their psychological distress [3,10]. However, judging from their characteristics and relations, they seem closer to the concept of workaholics [27-29]. Nevertheless, this addiction is associated with burnout [30], and may be one of the possible causes of it [31,32] due to exhaustion of the individual's energy resources. Highly committed subjects typically show a great likelihood of developing burnout [2,5], as their commitment and addiction are related by means of the absorption factor [30], making the employee a captive of his or her own activity [33-35]. Consequently, by learning to keep a certain distance from work and prioritising self-care, individuals could avoid excessive involvement and prevent burnout [36].

The underchallenged profile comprised the indifference, lack of development and boredom dimensions. This last factor, despite fulfilling Kaiser's criteria, presented a low percentage of explained variance, likely due to its high association with the other two factors. However, this factor should be included in the model because its content clearly differs from that of the other two factors. We observed relations between the underchallenged profile and exhaustion, lack of efficacy and, particularly, cynicism. Underchallenged employees have lost interest in the tasks involved in their work, have become cynical, and consequently seem to be affected by preliminary stages of burnout, such as dissatisfaction, limited variety and absence of feedback in tasks [15,37]. In other works, it has been observed that individuals' perception that other jobs would better acknowledged their talents, lack of interest or gratification, and monotony could precede burnout [11,13-15]. Specifically, the perception of minimum likelihood of personal development in a job predicts burnout in three years [38]. Efforts aimed at increasing employees' personal and career development and reducing boredom and apathy appear to lower levels of stress and exhaustion [39].

The worn-out profile is characterised by neglect, lack of control and lack of acknowledgement. The low scores and lower variability for items belonging to the neglect factor suggest that social desirability may have influenced subjects' responses. The worn-out type presents significant relations with exhaustion, cynicism and lack of efficacy, and therefore appears to be the profile that best fits the definition of burnout provided by Maslach, Schaufeli and Leiter [1]. Their neglect and/or apathy are associated with a lack of efficacy and may be inversely related to drive, participation and absorption [40], aspects considered diametrically opposed to burnout [41]. The desperation caused by absence of control over results has been related to high levels of stress, exhaustion, emotional fatigue and depersonalisation [38,39,42,43], which is in line with our results. The current study also shows that the perception of lack of acknowledgement is strongly associated with cynicism. Moreover, this appears to produce dissatisfaction and burnout in general [44]. Greater acknowledgement seems to have a positive influence on the work climate of an organisation, reducing exhaustion and raising quality of life at work [44,45].

Structural conditions, such as the temporary nature of work contracts, accentuate the development of some types of burnout. According to our results, temporary employees exhibit significantly higher scores for the frenetic subtype, associated with excessive dedication. Permanent employees displayed significantly higher scores for the underchallenged and worn-out subtypes, characterised by lower dedication. Significant differences were also found in the involvement, ambition, indifference, boredom, lack of acknowledgement and neglect dimensions, with the first two being higher in temporary workers, and the remaining dimensions higher in permanent employees. The structural condition of the temporary nature of work contracts appears to be associated with the type of burnout experienced, perhaps owing to differential involvement in work tasks. On the contrary, there were no significant differences by gender.

Although the characteristics of the subtypes may comprise determining factors for burnout syndrome, not all profiles fit the definition of Maslach, Schaufeli and Leiter [1] in the same way. These results can be explained if we interpret the burnout subtypes as different stages in the development of the syndrome, as proposed by Montero-Marín et al. [10]. The development of burnout syndrome is arranged longitudinally by degree of dedication at work, which progresses from more to less (from enthusiasm to apathy) [5,10,46,47]. Therefore, burnout appears to develop at a time of excessive involvement and commitment, typical of the frenetic profile [2,5,10,38]. Given that it is not easy to maintain this level of activity without becoming exhausted or affected [31], workers will adopt a certain distancing to protect themselves, behaving with indifference and cynicism [48,49]. While alleviating excess activity owing to excessive involvement, this distancing produces the type of frustration and stress suffered by the underchallenged profile [50]. Distancing also erodes the perception of efficacy in the long run by leading to passive coping strategies, such as neglect of responsibilities and emotional venting, which are typical of the worn-out profile [51-55].

Subtypes are affected by different sources of stress and discontent at work, depending on the level of dedication with which they cope with obstacles and difficulties. Consequently, in order to efficiently adapt treatment strategies for burnout syndrome, we must specifically consider the burnout subtype experienced in each case. From a clinical perspective, exclusive consideration for the most recent manifestations of the syndrome, as performed in current evaluation standards, are insufficient. In order to overcome this limitation, it is necessary to have a more extensive definition for burnout syndrome that takes into account the level of involvement with which subjects cope with their work as part of the syndrome development process.

This study has several limitations. First, a low response rate was obtained. However, the rate is quite similar to those found in previous studies using internet surveys [17,18]. This low rate could produce a bias in assessing point prevalence values, but does not affect the assessment of relationship patterns among different variables [17]. In addition, differences in response rates based on occupational level could decrease the representativeness of the sample; however, all of the various jobs showed the expected response rate values [17,18]. Another limitation is the sample selection, which was exclusively composed of workers from the University of Zaragoza. However, the sample was big and multi-occupational, as individuals in several jobs were included, improving the external validity of the study. Finally, this was exclusively a psychometric study; therefore, the predictive validity of the model has not yet been demonstrated. One of the main strengths of this study is that data quality was controlled by eliminating possible errors in the questionnaire transcription process through the use of purpose-designed software.

## Conclusions

The results of this study provide empirical support for the factor validity and internal consistency of the scales comprising the three clinical profiles. The Burnout Clinical Subtype Questionnaire is interesting in that it allows measurements for the three different burnout subtypes to be established. Moreover, it does so in a brief and operational manner, which makes it quite useful for the design and evaluation of specific treatment strategies for burnout syndrome.

## Competing interests

The authors declare that they have no competing interests.

## Authors' contributions

JM-M and JG-C are the principal researchers and developed the original idea for the study and the study design. JM-M developed the statistical methods. Both authors have read and corrected draft versions and approved the final version.

## Pre-publication history

The pre-publication history for this paper can be accessed here:

http://www.biomedcentral.com/1471-2458/10/302/prepub

## Supplementary Material

Additional file 1 **Appendix**. Burnout Clinical Subtype Questionnaire Click here for file
