# Supplementary material for: A newer and broader definition of burnout: Validation of the "Burnout Clinical Subtype Questionnaire (BCSQ-36)"
Source: BMC Public Health. 2010 Jun 2;10:302. doi: 10.1186/1471-2458-10-302 (PMC2887826; doi:10.1186/1471-2458-10-302)
Supplement: Additional file 1 — Appendix. Burnout Clinical Subtype Questionnaire [file 1471-2458-10-302-S1.DOC]

**APPENDIX**

**“Burnout Clinical Subtype Questionnaire” (BCSQ-36)**

The following is a series of statements indicating experiences that may occur at work. Read each statement carefully and mark with an X the option that best represents how you feel, what you do and what you think about your work. There are no right or wrong answers. Please **DO NOT LEAVE ANY STATEMENT UNANSWERED.**

|  | **Totally disagree** | **Strongly disagree** | **Disagree** | **Unsure** | **Agree** | **Strongly agree** | **Totally**  **agree** |
| --- | --- | --- | --- | --- | --- | --- | --- |
| 1. I need to achieve great success in my work | O | O | O | O | O | O | O |
| 2. I think the dedication I invest in my work is more than what I should for my health | O | O | O | O | O | O | O |
| 3. I invest the necessary effort in my work until I overcome difficulties | O | O | O | O | O | O | O |
| 4. I am ambitious to obtain important results in my work | O | O | O | O | O | O | O |
| 5. I neglect my personal life when I pursue important achievements in my work | O | O | O | O | O | O | O |
| 6. I get very involved in solving work-related problems | O | O | O | O | O | O | O |
| 7. I feel the need to achieve important goals in my work | O | O | O | O | O | O | O |
| 8. I risk my health when I pursue good results in my work | O | O | O | O | O | O | O |
| 9. If I don’t achieve the expected result in my work, I try harder to achieve it | O | O | O | O | O | O | O |
| 10. I have a strong need for important achievements in my work | O | O | O | O | O | O | O |
| 11. I overlook my own needs to fulfil work demands | O | O | O | O | O | O | O |
| 12. I react to difficulties in my work with greater participation | O | O | O | O | O | O | O |
| 13. I feel indifferent about my work and have little desire to succeed | O | O | O | O | O | O | O |
| 14. I would like to be doing another job that is more challenging for my abilities | O | O | O | O | O | O | O |
| 15. I feel my work is mechanical and routine | O | O | O | O | O | O | O |
| 16. I have little interest for the tasks involved in my job | O | O | O | O | O | O | O |
| 17. I feel that my work is an obstacle to the development of my abilities | O | O | O | O | O | O | O |
| 18. My work offers me little variety in its activities | O | O | O | O | O | O | O |
| 19. I’m not enthusiastic about my work | O | O | O | O | O | O | O |
| 20. I would like to be doing another job where I can better develop my talents | O | O | O | O | O | O | O |
| 21. I am unhappy with my work because the tasks involved are monotonous | O | O | O | O | O | O | O |
| 22. I behave in a unconcerned and reluctant way at work | O | O | O | O | O | O | O |
| 23. My work doesn’t offer me opportunities to develop my abilities | O | O | O | O | O | O | O |
| 24. I feel bored at work | O | O | O | O | O | O | O |
| 25. The people who need my services don’t show appreciation or gratitude for my efforts | O | O | O | O | O | O | O |
| 26. When things at work don’t turn out as well as they should, I stop trying | O | O | O | O | O | O | O |
| 27. I feel helpless in many situations in my work | O | O | O | O | O | O | O |
| 28. Professional recognition doesn’t depend on efforts made at work | O | O | O | O | O | O | O |
| 29. I give up in response to difficulties in my work | O | O | O | O | O | O | O |
| 30. I feel defenseless in some situations in my work | O | O | O | O | O | O | O |
| 31. The organisation I work for doesn’t take notice of effort and dedication | O | O | O | O | O | O | O |
| 32. I give up in the face of any difficulties in my work tasks | O | O | O | O | O | O | O |
| 33. I feel the results of my work are beyond my control | O | O | O | O | O | O | O |
| 34. I think my dedication to my work is not acknowledged | O | O | O | O | O | O | O |
| 35. When the effort I invest in work is not enough, I give in | O | O | O | O | O | O | O |
| 36. I deal with many situations in my work that are beyond my control | O | O | O | O | O | O | O |
